# Local adaptation and colonization are potential factors affecting sexual competitiveness and mating choice in *Anopheles coluzzii* populations

**DOI:** 10.1038/s41598-021-04704-8

**Published:** 2022-01-12

**Authors:** Charles Nignan, Bèwadéyir Serge Poda, Simon Péguédwindé Sawadogo, Hamidou Maïga, Kounbobr Roch Dabiré, Olivier Gnankine, Frédéric Tripet, Olivier Roux, Abdoulaye Diabaté

**Affiliations:** 1 Institut de Recherche en Sciences de La Santé (IRSS), Bobo-Dioulasso, Burkina Faso; 2 Laboratoire d’Entomologie Fondamentale Et Appliquée, Unité de Formation Et de Recherche en Sciences de La Vie Et de La Terre (UFR-SVT), Université Ouaga I Pr. Joseph KI-ZERBO, Ouagadougou, Burkina Faso; 3 MIVEGEC, IRD, CNRS, University of Montpellier, Montpellier, France; 4 Centre for Applied Entomology and Parasitology, School of Life Sciences, Keele University, Keele, UK

**Keywords:** Ecology, Evolution, Zoology

## Abstract

The mating behaviour of the malaria vector *Anopheles gambiae* complex is an important aspect of its reproduction biology. The success of mosquito release programmes based on genetic control of malaria crucially depends on competitive mating between both laboratory-reared and wild individuals, and populations from different localities. It is known that intrinsic and extrinsic factors can influence the mating success. This study addressed some of the knowledge gaps about factors influcencing mosquito mating success. In semi-field conditions, the study compared the mating success of three laboratory-reared and wild allopatric *An. coluzzii* populations originating from ecologically different locations in Burkina Faso. Overall, it was found that colonization reduced the mating competitiveness of both males and females compared to that of wild type individuals. More importly, females were more likely to mate with males of their own population of origin, be it wild or colonised, suggesting that local adaptation affected mate choice. The observations of mating behaviour of colonized and local wild populations revealed that subtle differences in behaviour lead to significant levels of population-specific mating. This is the first study to highlight the importance of local adaptation in the mating success, thereby highlighting the importance of using local strains for mass-rearing and release of *An. coluzzii* in vector control programmes.

## Introduction

Local adaptation is an evolutionary process whereby species acquire traits that provide an advantage under local environmental conditions, regardless of the consequences of those traits for fitness in other habitats^1^. Thus, ‘local’ populations are defined as a limited area where an isolated population has been under selection pressure, adapting to specific aspects of the environment^1^. Consequently, local adaptations may affect classical biological control strategies^2^ designed to reduce the population size of a local pest by the introduction of natural enemies from a different area. Similarly, it can affect genetic vector control approaches such as the Sterile Insect Technique (SIT), which consists of the release of a large number of sterile males into a wild local population to mate with wild females, resulting in non-viable progeny and consequently a reduction of the targeted population size^3^. However, both local adaptation and/or insect rearing in the laboratory can alter an insect's ability to interact and affect its reproductive compatibility^4^.

Mosquitoes from the *Anopheles gambiae* complex are the most important vectors of malaria in sub-Saharan Africa^5^. Their capacity to adapt to diverse environments allows their proliferation in semitropical, tropical and desert climates, and has contributed to speciation within the complex^6,7^. Current vector control programmes rely heavily on chemical control using Long Lasting Insecticide treated Nets (LLINs) and Indoor Residual Spraying (IRS). The efficacy of these tools is decreasing because of the spread of insecticide resistance in the major malarial vector species, which can jeopardize the success of malaria control programmes^8^.

Consequently, over the last two decades, researchers have increasingly focused their attention on mosquito mating behaviour to enhance the efficacy of sterile male release procedures to control vector populations and the pathogens they transmit^9^. Mating in the *An. gambiae* s.l. species complex occurs in swarms, and swarming activity has a particularly high energy cost^10^. Males chase virgin females with the aim of forming a copulae^11^. Several studies have shown that the male mosquito’s flight ability is negatively affected by laboratory colonization and subsequent multi-generational rearing^12,13^. Colonised males seem to adapt to laboratory conditions where they expend low amounts of energy during mating activities as there is little selective pressure to mate quickly in small rearing cages^14^. Successful implementation of vector control approaches depends on effective mating by mass-reared and released individuals. Therefore, it is essential to understand how the processes of mate choice is affected in males and/or females when they are from different populations, i.e. from laboratory and wild populations and from allopatric populations^15,16^.

Some studies have suggested that long-range windborne dispersal in *An. coluzzii* might result in gene flow between distant populations^17,18^. However, large genotypic and phenotypic differences between populations can be detected^19–23^ which strongly suggest the existence of local adaptations. Understanding whether local adaptations negatively affect mating fitness of long distance dispersers is key to assessing whether this can translate into effective mating and gene flow. While numerous studies tested effects of laboratory rearing on mosquito mating compatibility^12,13,24^, few attempted to test mating compatibility of wild populations^25–27^.

Given the potentially negative impact of local adaptations on efforts to deploy SIT on a large scale, this study aimed to assess whether local adaptation—driven either by environmental conditions in wild populations or by rearing conditions—influenced mating competitiveness and mate choice among three wild populations and between a laboratory population and its original wild source population.

## Results

### Experiment 1: Effects of colonisation on swarm characteristics and mating success

The origin of males (laboratory vs. wild) had no effect on swarm size with a estimated mean number of mosquitoes in the swarm of 59 ± 6 out of 300 released males ($\chi_{1}^{2}$ = 0.08, *P* = 0.8). However, laboratory mosquitoes formed swarms which flew higher than their field counterparts ($\chi_{1}^{2}$ = 4.2, *P* = 0.03, 157 ± 4 cm *vs*. 140 ± 6 cm, respectively). Neither the swarm size nor the interaction between male origin and swarm size had any effect on swarm heights $(\chi_{1}^{2}$ = 1.1, *P* = 0.3; $\chi_{1}^{2}$ = 0.69, *P* = 0.4, respectively).

**Figure 1:**
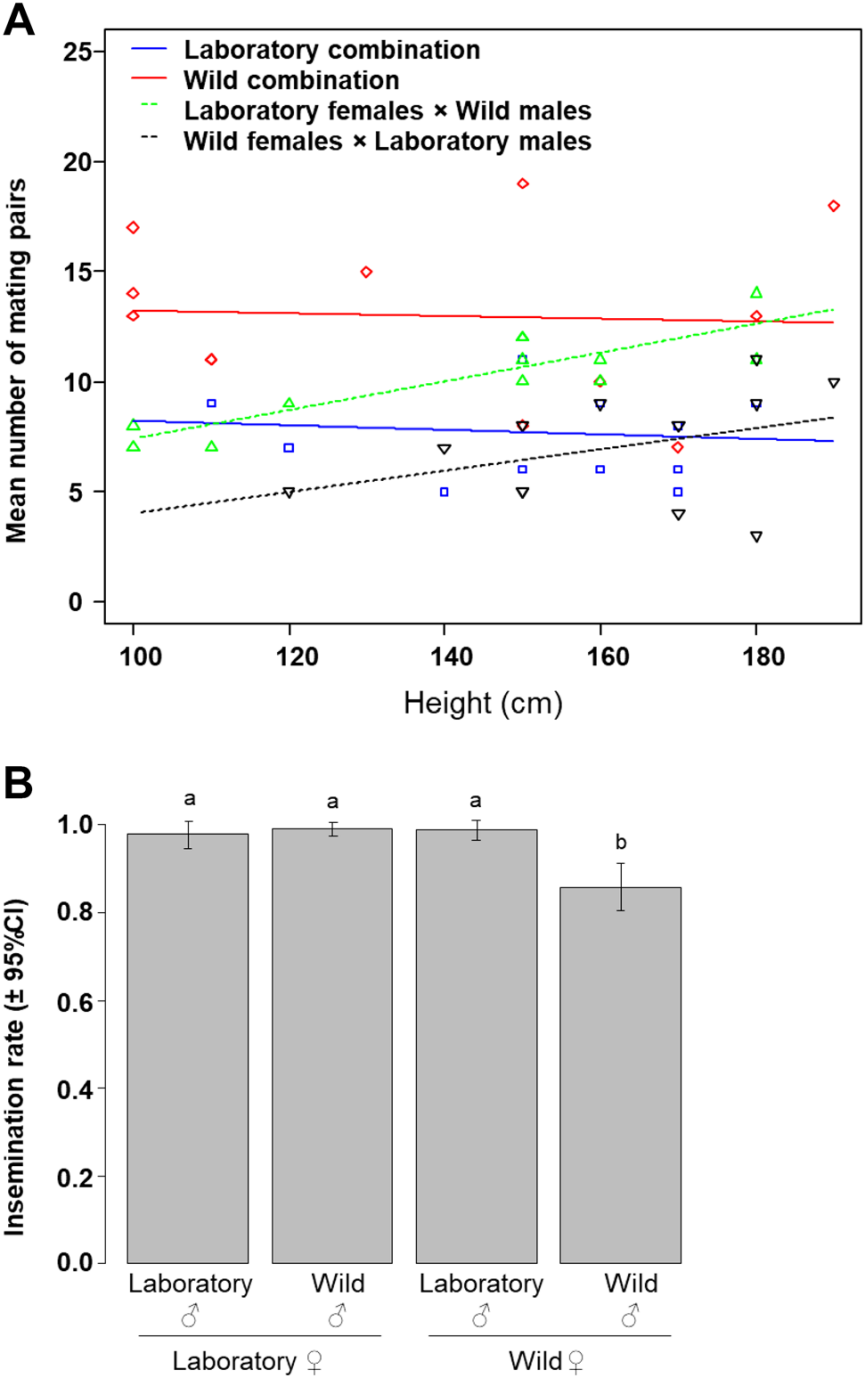
Effects of colonization on swarm characteristics and mating success (Experiment 1). (**A**) Mean number of mating pairs according to mating combinations and swarm height; (**B**) Mean insemination rate. CI: 95% confidence interval. SE: standard errors. Different letters above bars show statistical significance for multiple comparisons at *P* < 0.05.

A total of 454 mating pairs were collected. The interaction between mating combinations and swarm heights was marginally significant ($\chi_{3}^{2}$ = 7.98, *P* = 0.04), with a relatively stable number of mating pairs in the control mating combinations whatever the swarm height, while the number of mating pairs slightly increased in mixed mating combinations as swarm height increased (Fig. 1A). All other interactions and factors had no effects on the number of mating pairs collected (mating combination*swarm height*swarm size, $\chi_{3}^{2}$ = 3.8, *P* = 0.27; swarm size*swarm height, $\chi_{1}^{2}$ = 0.02, *P* = 0.8; mating combinations*swarm size, $\chi_{3}^{2}$ = 3.9, *P* = 0.26; swarm size, $\chi_{1}^{2}$ = 0.41, *P* = 0.5).

An overall insemination rate of 94.27% was obtained from females collected in mating pairs. There was a significant effect of mating combinations on insemination rate ($\chi_{3}^{2}$ = 29.2, *P* < 0.001, Fig. 1B), with females of the wild control mating combination being less likely to be inseminated in our semi-field conditions.

### Experiment 2: Effects of colonisation on sexual competitiveness

**Figure 2:**
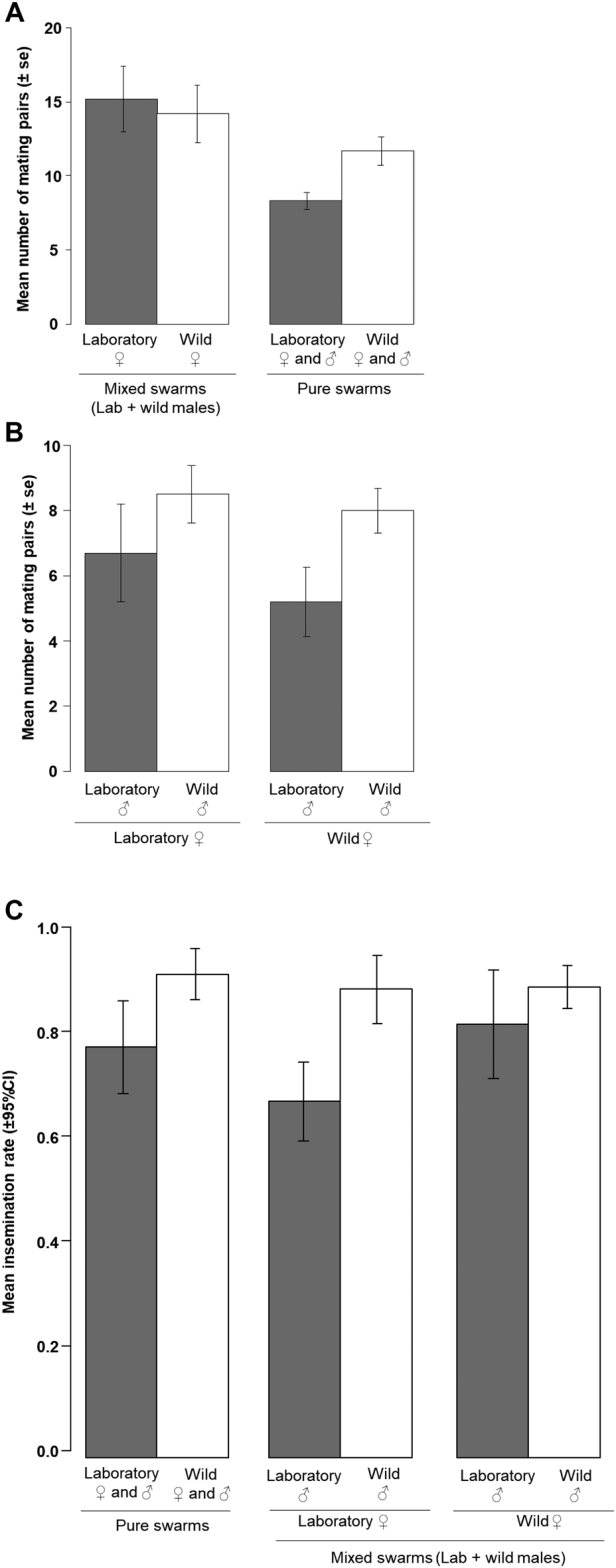
Effects of colonisation on male competitiveness (Experiment 2). (**A**) Mean number of mating pairs in both pure and mixed swarms of laboratory and wild males; (**B**) Observation of assortative mating through the mean number of mating pairs in mixed swarms with laboratory or wild females; (**C**) Mean insemination rate. CI: 95% confidence interval. SE: standard errors.

In experiment 2, both wild and laboratory males were simultaneously released with females to determine whether there was a difference in their ability to compete for females. A total of 846 mating pairs were collected from all the tested mating combinations. There was a significant interaction between swarm type (pure or mixed) and female origin on the number of mating pairs ($\chi_{1}^{2}$ = 5.47, *P* = 0.02; Fig. 2A). Specifically, mixed swarms produced more mating pairs than pure swarms whatever the female origin, while pure laboratory swarms produced less mating pairs than pure wild swarms (with their respective females). There was no effect of the interaction between swarm size and swarm type ($\chi_{1}^{2}$ = 0.39, *P* = 0.5) or the swarm size alone ($\chi_{1}^{2}$ = 2.9, *P* = 0.08) on the number of mating pairs collected. However, in mixed swarms, wild males were more prone than laboratory males to mate with either wild or laboratory females ($\chi_{1}^{2}$ = 7.9, *P* = 0.004) (Fig. 2B). On the other hand, neither the female origin nor the interaction between the male origin and female origin had effects on the number of mating pairs ($\chi_{1}^{2}$ = 0.65, *P* = 0.4; $\chi_{1}^{2}$ = 0.46, *P* = 0.4, respectively).

An overall insemination rate of 94.42% was obtained from females collected in mating pairs. There was a significant effect of swarm type on insemination rate ($\chi_{1}^{2}$ = 5.3, *P* = 0.02; Fig. 2C) with a greater proportion of females being inseminated in pure swarms than in mixed swarms (0.85 ± 0.05 and 0.79 ± 0.05, respectively). The mating combination also had a significant effect on insemination rate ($\chi_{3}^{2}$ = 20.4, *P* = 0.0001; Fig. 2C). The highest insemination rate was recorded in wild females mated to wild males (0.92 ± 0.04). Laboratory females mated with laboratory males showed the lowest rate of insemination (0.55 ± 0.11). However, no interaction was found between mating combination and swarm type on insemination rate ($\chi_{1}^{2}$ = 0.8, *P* = 0.3).

### Experiment 3: Swarm characteristics and male competitiveness of three wild populations.

**Figure 3:**
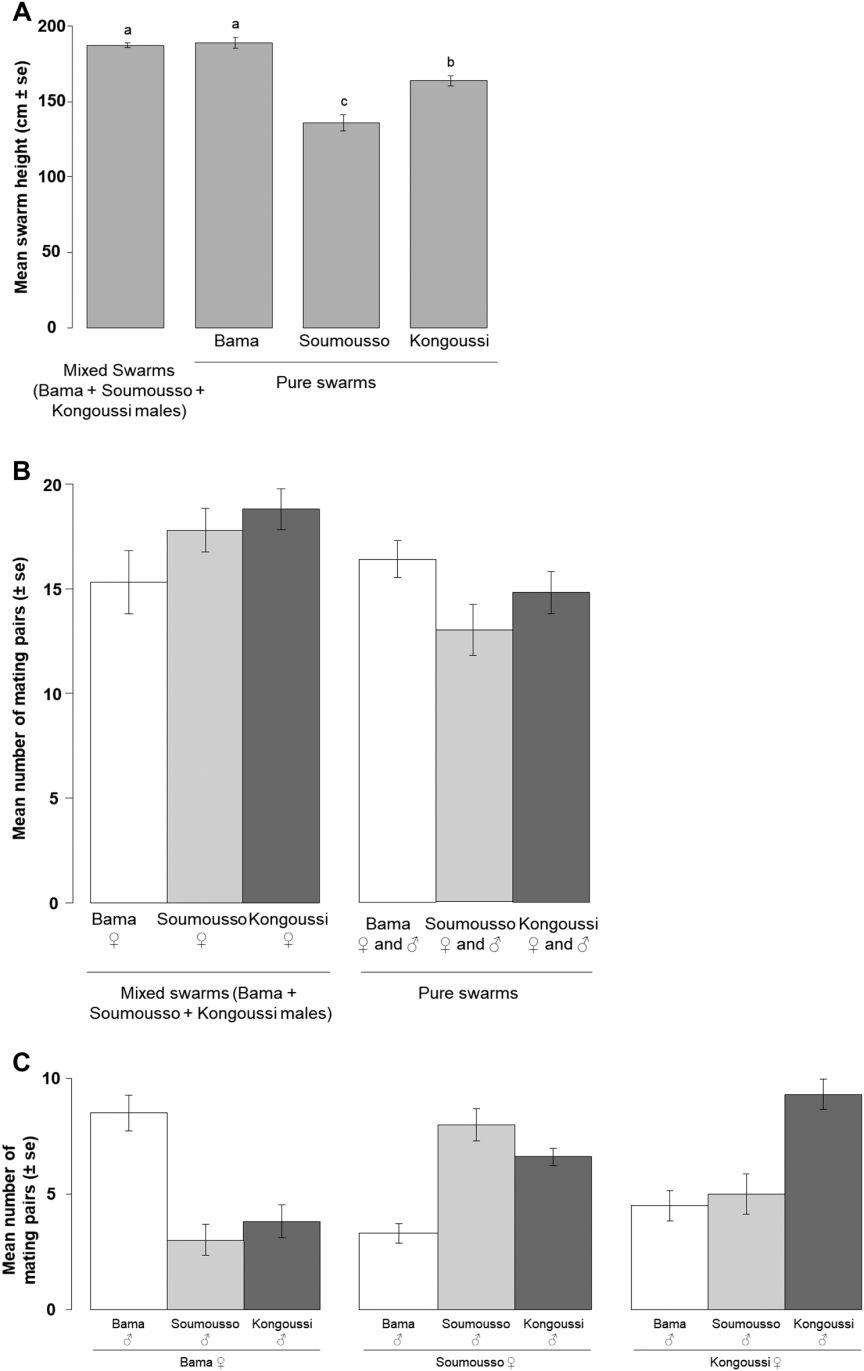
Swarm characteristics and male competitiveness in three wild populations (Experiment 3). (**A**) Mean swarm height in each population and in mixed swarms; (**B**) Mean number of mating pairs produced in both pure and mixed swarms with females of the three populations; (**C**) Observation of assortative mating through the mean number of mating pairs obtained in mixed swarms with females of each population. SE: standard errors. Different letters above bars show statistical significance for multiple comparisons at *P* < 0.05.

Swarm centroids (i.e. the location where the density of mosquitoes in the swarm was the highest) were typically observed between 100 and 300 cm high above the cloth marker placed on the ground. The geographic origin of males had a strong effect on swarm height ($\chi_{3}^{2}$ = 69.1, *P* < 0.001). Males from Bama swarmed higher than those from Kongoussi, while those from Soumousso swarmed at the lowest height (Bama 189 ± 3 cm, Kongoussi 164 ± 3 cm, Soumousso 136 ± 5 cm; Fig. 3A). Mixed swarms flew at a similar height to those of Bama and higher than those of Soumousso or Kongoussi. Indeed, the mixed swarm did not produce the usual single centroid. Two centroids were seen above the marker, one larger than the other. However, neither swarm size nor the interaction between swarm size and swarm type had any effect on swarm height ($\chi_{1}^{2}$ = 1.74, *P* = 0.18; $\chi_{3}^{2}$ = 7.03, *P* = 0.07, respectively).

A total of 965 mating pairs were collected from the tested mating combinations. There was a significant interaction between swarm type and female origin on the number of mating pairs ($\chi_{2}^{2}$ = 9.9, *P* = 0.007; Fig. 3B). More mating pairs with females from Kongoussi and Soumousso were collected from mixed swarms, whereas similar numbers of mating pairs were collected from both mixed and pure swarms with females from Bama. Both swarm size and the interaction between swarm size and swarm type had no effect of the number of mating pairs ($\chi_{1}^{2}$ = 0.17, *P* = 0.7 and $\chi_{1}^{2}$ = 1.5, *P* = 0.2, respectively). In mixed swarms, mating between males and females from the same origin were more likely than between two different origins ($\chi_{1}^{2}$ = 52, *P* < 0.001; Fig. 3C). However, the following factors had no effect on the number of mating pairs: female origin; swarm size; interaction between swarm size and mating choice; interaction between mating choice and female origin ($\chi_{1}^{2}$ = 4.4, *P* = 0.1; $\chi_{1}^{2}$ = 1.6, *P* = 0.2; $\chi_{1}^{2}$ = 0.67, *P* = 0.4; $\chi_{1}^{2}$ = 3.3, *P* = 0.2, respectively).

An average insemination rate of 96.26% was found. Despite assortative mating within mixed swarms, there was no evidence of difference in insemination rates (swarm type*mating pair identity $\chi_{2}^{2}$ = 1.1, *P* = 0.5; swarm type: $\chi_{1}^{2}$ = 0.7, *P* = 0.4; mating pair identity: $\chi_{8}^{2}$ = 7, *P* = 0.5).

## Discussion

This study assessed the effects of colonization and locale adaptation on *An. coluzzii* mosquito swarming behaviour, mating compability, competitiveness between laboratory-reared and wild populations, and three different wild popualtions. The results showed that colonization negatively infuenced the mating competitiveness and females were more likely to mate with males of their own population of origin, be it wild or colonised. This suggested that local adaptation affected mate choice.

Previous studies reported that colonisation affects mosquito swarming behaviour^10,26,28^ and showed that behaviours of wild populations can be lost under laboratory conditions because colonized individuals are not subject to the same challenges as those in the field^24,29^. The success of sterile insect technique relies primarily on male mosquitoes produced in a laboratory or rearing factory, after their release onto the wild, being able to survive, locate a swarming spot and compete with wild males for wild females. The results of this study show that the conditions in which mosquitoes were reared in the laboratory affected some swarm characteristics and male mating abilities. Indeed, laboratory males swarmed at about 17 cm higher than wild males. Previous studies have shown that *An. coluzzii* males swarm at ~ 200 cm above the ground in the field^30^, and that field *An. coluzzii* swarmed between 120 and150 cm in our semi-field setup^31^. Swarm height potentially plays an important role in the mating biology of *Anopheles* mosquitoes. In the absence of males, females display the same swarming behaviour as their respective males flying at the same location and at the same height^31^. Therefore, we can hypothesize that if the difference in male and female swarm heights is too large, the chance of their meeting each other could be reduced. In this study the height at which the male density was the highest (the centroid) was measured, but did not take into account the total vertical amplitude occupied by the swarm. Poda et al*.* (2019) described that, in semi-field cages, the amplitude of height of *An. coluzzii* male swarms was distributed between 50 and 200 cm (with mean minimal and maximal heights of 107 and 176 cm, respectively). Consequently, the 17 cm difference between wild and laboratory swarm centroids was probably not biologically relevant and wild females would probably still encounter laboratory males with similar frequencies. Nevertheless, it was observed that laboratory males were less efficient at mating with females in the swarms (lower number of couples). However, they inseminated a higher rate of wild females than wild males. These results suggest that laboratory males have undergone rapid and profound changes to adapt to laboratory mating conditions, which are generally confined spaces, high densities, 1:1 sexual ratios and matings without swarm formation. These conditions are very different from or even opposite to the natural mating conditions. Therefore, adapting to these mating conditions means being less able to mate in natural conditions. Evidence of adaptation to laboratory conditions has been observed in *An. coluzzii* strains (KIL strain) which have been laboratory reared for more than 35 years^32^. In this case, KIL males were capable of inseminating more females per night than wild type males in laboratory conditions. Moreover, other studies have also shown that body size and inbreeding could affect male *An. coluzzii* reproduction^32,33^. Unfortunately, there was no measurement of mosquito size to check for any correlation with mosquito origin and/or mating ability. This can be considered as a limitation of the study that could be further investigated. In addition, mosquito colonies continuously lose genetic variation over time^32^. Such changes in genetic composition of laboratory colonized populations are also often accompanied by appearance of undesired phenotypic^13^ and behavioural traits, such as poor mating ability^34,35^. Therefore, these results suggest that colonisation may affect swarming behaviour, but laboratory males are still able to mate with and inseminate wild females.

If laboratory mosquitoes are released into the wild, they will need to compete with their wild counterparts in the same swarms. Consequently, their ability to mate in mixed swarms was assessed. The results show that mixed swarms produced more mating pairs than pure swarms. Based on the data, this result is difficult to explain, but two nonexclusive hypotheses may be stated. First, the increased number of couples could be linked to an increase in the number of females entering into swarms, but it is impossible to measure this trait by eye. This higher swarm attractiveness could be due to a greater swarm amplitude which would make the swarm more visible and provide it with a larger mating area. Unfortunately, in this study this trait was not measured. However, on some occasions, several centroids were observed in mixed swarms which could make them more stretched than the pure swarms. Second, exposing females to a greater choice of males (laboratory plus wild males) could increase the likelihood of females ecountering and accepting an "adequate" male.

In mixed swarms, wild males outcompeted laboratory males for wild females, again demonstrating that the laboratory rearing conditions altered the ability of males to acquire wild females. This could have been due to either a lower mating success or the previously mentioned height at which laboratory males fly in swarms. If the latter was true, laboratory males should perform better than wild ones when in contact with laboratory females. However, it was observed that wild males had similar mating competitiveness with laboratory males and were sometimes better, when mating with laboratory females. Thus, the most likely explanation is that colonization of male mosquitoes alters mating characteristics resulting in compromised mating success under semi-field and probably under field conditions^36,37^. Similarly, a study carried out in Mali showed that *An. coluzzii* wild males mated at higher frequency with both conspecific wild and laboratory females than laboratory males^12^. In contrast, in *Aedes*, another mosquito genus, the mating competitiveness of two RIDL strains of *Aedes aegypti*, expressing a repressible dominant lethal gene, successfully competed with males from a local wild-type of *Ae. aegypti* in large laboratory cages in semi-field conditions^26^.

The results of the present study show that in mixed swarms, wild males outcompeted laboratory males for wild females when mating in a contained semi-field system. In Experiments 1 and 2, no significant difference was found in the insemination rate of wild females mated with either laboratory or wild males in competition with each other or not. However, the insemination rates might be overestimated by the fact that male and female of each couple were kept together overnight after collection at dusk and then analyzed the following morning. Nevertheless, the mosquito mating success is fitness dependent^12^. Therefore, the wild and laboratory males demonstrated the same fitness levels when competing for females, thus indicating a sexual compatibility that led to wild females being mated frequently with laboratory males. Similar results have been reported in *An. coluzzii*, for which no difference in insemination rates were found between wild and laboratory strains^12^. Moreover, in another study, irradiated *An. arabiensis* laboratory males mated more with wild females than with laboratory females^38^. This may be explained by a likely consequence of a reduced genetic variation generally inherent in laboratory colonies as compared to the wild populations from which they originated. Adapation to laboratory-rearing conditions could make colonised mosquitoes less demanding when chosing a mate: laboratory males would copulate with both laboratory and wild females while wild males would reject mating with laboratory females due to their unnatural mating behaviours. Moreover, several swarm centroids observed in the mixed swarms, suggested that the both populations would not form a swarm as a unity but two merged swarms. The mosquito populations would swarm at different heights in the swarm to mate assortatively.

In biological control strategies based on insect releases, released and target populations are usually from different localities. Mating compatibility between the wild populations is essential and should be the first of things to assess. To assess if potential local adaptations in the reproductive behaviour occur in different wild populations of *An. coluzzii*, swarming behaviour and mating abilities in both pure and mixed swarms of three wild populations were investigated. As expected, the males of the three localities (Bama, Soumousso and Kongoussi) located their swarm above the visual marker. However, the swarm heights were significantly different among populations, with the greatest difference in the centroid height of 53 cm between Bama and Soumousso populations, which are also the two geographically closest populations in our study. Even if it is a difference between the centroids and not the swarm amplitude, this difference could be biologically significant in small swarms. If no cues other than the visual markers are used to locate swarming sites, "local" females may miss small swarms formed by "introduced" males. Such differences have also been observed in the wild between distant populations of *An. coluzzii* with a centroid measured at 2 m in Bama, Burkina Faso^30^ and 0.7 m in Djègbadji in Benin^39^. Nevertheless, swarm height is also influenced by the marker size^31^ and probably also by environmental conditions or visual cues that can alter the horizon^40,41^.

When the three populations swarmed above the swarm marker, a single swarm was observed when the three populations were released together. As in the experiment with laboratory-reared and wild popultions, several centroids were frequently observed and the most dense one was also the highest one at a height similar to the Bama centroids. The reason why the centroid of mixed swarms corresponded to the highest centroid height among the three populations is unknown. Swarm centroids are thought to be an advantageous location for males to intercept females seeking a mate because they can quickly reach any sector of the swarm^42^, but maybe also because females swarm at a similar height to males^31^ which increases the likelihood of encounters.

The observation of several centroids needs further investigation. Although they were observed in mixed swarms only, it is unknown whether it is the result of a compartmentalization according to the male origins or whether there were several competition hotspots with "dominant" males in the largest centroids and less dominant males in the smaller ones. This last hypothesis is less likely as multiple centroids were not observed in pure swarms. Capture of males at different heights and recordings of the height at which females of different origins enter into the swarm, or at least the height at which mating pairs form, could provide valuable information.

As in our experiments focusing on the mating competitiveness of colonized laboratory *versus* wild males, mixed swarms produced more mating pairs than pure swarms and the same hypotheses as developed above can thus be stated. Consequently, in such conditions, it could be expected that the three populations would produce more mating pairs in mixed swarms than in pure swarms due to a greater swarm amplitude. Swarm amplitude which is correlated with swarm size and/or mosquito density could lead to a greater choice in males and a higher probability of encounter for species without assortative mating behaviour. However, this was not the case for the Bama population although both mixed swarms and Bama swarms shared the same centroid height and thus should have conferred an advantage to Bama females to find a mate. This raises the question of assortative mating in mixed swarms. Our results have shown that in all the mixed swarms, about 50% of matings were assortative against ~ 25% for each of the two other non-assortative mating combination. This suggests either a vertical swarm compartmentalization with males of each population occupying a species specific height at which the females of their respective population will search for a mate and thus increasing the likelihood to mate assortatively; or the existence of recognition mechanisms allowing males and females to discriminate according to their origin during close-range interaction due to acoustic cues^43–46^ or contact chemical cues which are under both genetic and environmental control^47^. Assortative mating between individuals from the same population suggests that control programmes based on male release should rear and release individuals that are closely related to the targeted population. For programmes covering large geographical areas, it would imply that the number of rearing strains need to be multiplied. An alternative solution would be to increase the number of males released to counterbalance the negative effect of assortative mating.

The success of biological control strategies based on mating behaviour requires knowledge of the ecology of the target population and the ability of the released population to adapt to local conditions, find its target and successfully mate with it. The two major potential weak points of these strategies are the effect of laboratory rearing and local discriminations. Our results have shown that in *An. coluzzii* from Burkina Faso, both our rearing conditions and local differences in some swarming traits could be responsible for a partial lack of compatibility between the different populations tested. Laboratory males released for a strategy based on population sterilisation or genetic control have shown a lower capacity to mate with wild females compared to equivalent wild males. In SIT or other genetic control contexts, it means that a greater number of sterilized males would be necessary to achieve an effective reduction of the wild population or that the gene of interest could take more time to spread through the natural population. Assortative mating due to local adaptation of the mating phenotype was an important determinant of mating success in mixed swarms. In the field, male release programmes could encounter significant problems, resulting in limited mating with wild females and a potential failure of the control strategy. The proximate determinants resulting in evolution and maintenance of such local adaptations in *An. coluzzii* are unknown and need to be investigated.

## Methods

### Mosquito origins

**Figure 4:**
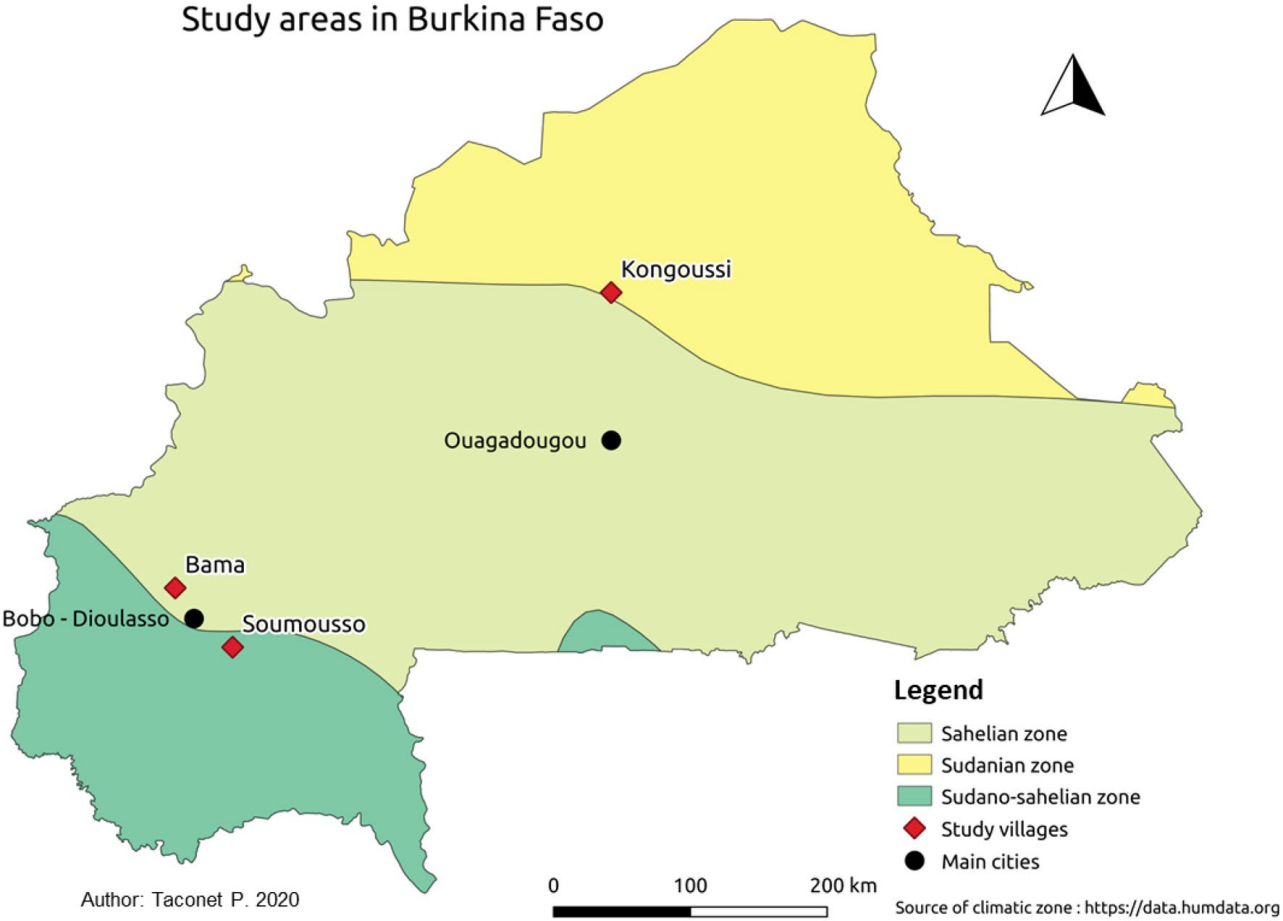
Location of collection points in Burkina Faso. The map includes the villages of the study with their climatic zones. Background of the map was produced with open data from openstreetmap.org. Data of climatic zones are obtained through: https://data.humdata.org/.

All experiments were performed with populations of *An. coluzzii* collected in three localities in Burkina Faso; Bama, where this species is prevalent, and Soumousso and Kongoussi, where *An. coluzzii* is found in sympatry with *An. gambiae* and *An. arabiensis*. Bama (4° 24′ W; 11° 24′ N) is located 30 km northwest of Bobo-Dioulasso (the second largest city of the country and, where our laboratories were located and mosquito breeding was carryied out) and is characterized by rice fields to the south and by savannah to the north. The irrigation system of rice fields forms permanent mosquito breeding sites in which *An. coluzzii* thrives all year. Soumousso village (4° 02′ W; 11° 00′ N) is located at 55 km south-east of Bobo-Dioulasso in the Soudanian zone, and has the typical Guinean savannah habitat. In this area, the rainy season extends from May to November with average yearly rainfall above 900 mm/year^8^. Kongoussi (1° 30′ W; 13° 17′ N) is located in the Sahelian zone, 118 km north from Ouagadougou (the country's capital) and has a shorter rainy season than the other sites, extending from June to September, with an average yearly rainfalls of 20–160 mm/year^8^. The Bam lake extends into the Kongoussi village providing year long mosquito breeding sites. The distances between the sampling locations are as follows: 375.33 km between Bama and Kongoussi; 376.84 km between Soumousso and Kongoussi; and 57.2 km between Bama and Soumousso (Fig. 4).

### Mosquito rearing and experimental settings

#### Laboratory colony

A laboratory colony of *An. coluzzii* was established in August 2012 from blood-fed females collected in human dwellings in Bama (VK7, a local area of Bama village), and was maintained in the lab for ca. 66 generations. The colony has been maintained in the insectary at the Institut de Recherche en Sciences de la Santé (IRSS) in Bobo-Dioulasso under standard conditions of temperature, relative humidity and light cycle (28 ± 1 °C, 80 ± 10% RH and 12:12 L:D). The larvae were reared in tap water and fed with Tetramin^®^ Baby Fish Food (Tetrawerke, Melle, Germany) ad libitum, and adults were placed in typical lab rearing cages (30 × 30 × 30 cm) and fed with a 10% glucose solution^48^.

#### Wild mosquitoes (F1 progeny)

Blood-fed *An. gambiae* s.l. females were collected in human dwellings in the three study areas, taken to the Mosquito Ecology Research Facility (MERF) located in Bama^49^ and placed in individual cups for egg laying. After eggs were laid, females were identified according to their species by Polymerase Chain Reaction^50^. The larvae of female *An. coluzzii* were pooled as per origin. The Larvae were reared at densities of about 400 larvae in plastic trays (30 cm × 20.5 cm × 6.5 cm) containing rain water and fed with Tetramin Baby Fish food (Tetrawerke, Melle, Germany) ad libitum*,* with similar feed concentrations to those used by Vantaux et al.(2016) to produce mosquitoes of medium body size^51^. Pupae were transferred from the rearing trays to plastic cups (Ø = 45 mm, h = 85 mm) at densities of about 200 pupae by cup and placed into 30 cm × 30 cm × 30 cm mesh-covered cages for emergence. Adults were held in 30 cm × 30 cm × 30 cm mesh cages at the densities of about 500 mosquitoes per cage with 10% glucose solution under standard insectary conditions (27 ± 2 °C, 70 ± 5% relative humidity, 12:12 LD). All experiments were performed with virgin male and female mosquitoes. To prevent mating before their use, mosquitoes were separed by gender at pupal stage. Male and female pupae were separated under a binocular microscope based on pupal terminaliae morphology and placed separately in cages.

#### Mosquito ecology research facility (MERF)

All experiments were performed in the MERF^29^, which is composed of 11 outdoor experimental compartments measuring 10 m × 6 m × 4.5 m (L × W × H) each, with a floor made of concrete, walls of polyester net and a roof of a transparent polythene, ensuring climatic conditions were similar to the surrounding ambient conditions and optimal diffusion of daylight into every compartment. Before starting the experiments, a 1.5 m × 1.5 m black cloth was placed at the centre of each compartment, to be provide a visual swarm marker similar in size and contrast againt the concrete floor as for natural swarm-markers used by *An. coluzzii* mosquitoes in order to position their swarm,^31,49^.

### Experiment 1: Effects of colonisation on swarm characteristics and mating success

Four experimental compartments of the MERF were used simultaneously. At sunset (18:00), 3- to 4-day-old virgin males and females, either from Bama (wild mosquitoes) or from the laboratory (Bama strain originally), were released into the compartments at the same time using a 3:1 (male: female) sex ratio. Four mating combinations (one per compartment) were tested: (a) 300 wild males + 100 wild females (wild control); (b) 300 laboratory males + 100 laboratory females (laboratory control); (c) 300 laboratory males + 100 wild females (wild females) and (d) 300 wild males + 100 laboratory females (laboratory females). Swarm characteristics such as the number of mosquitoes in the swarm (swarm size) and the height of swarm (corresponding to the height of the centroid, i.e. the height at which mosquito density in the swarm was the highest), were estimated by eye and recorded 15 min after swarming activity started (i.e. when the swarm reached its highest mosquito density^30^) as described in our recent previous study^49^. During the whole swarming period, mating pairs (i.e. male–female couples) were collected in flight using sweep nets. The male and female of the mating pairs were placed separately into individually labelled plastic cups and brought into the laboratory. The following day, the insemination status of the females (presence/absence of spermatozoa) was assessed by dissecting their spermathecae under a stereomicroscope (400 × magnification). Twelve replicates were performed for each mating combination. For each replicate, the mating combinations were randomly assigned to the four compartments to avoid compartment bias.

### Experiment 2: Effects of colonisation on sexual competitiveness

The experimental design was similar to Experiment 1 with the exception of introducing a mixture of both wild and laboratory males into the mating combinations to assess competitiveness. The mating combinations were: (a) 300 wild males + 100 wild females; (b) 300 lab males + 100 laboratory females; (c) 150 wild males + 150 laboratory males + 100 wild females; and (d) 150 wild males + 150 laboratory males + 100 laboratory females. Prior to release, males were marked in the morning of the test with one of two colours of fluorescent powder (Bioquip products 2321 Gladwick Street Rancho Dominguez, CA 90,220, USA; Ref: 1162 B, R, and Y) according to their origin (laboratory or wild). Fifteen minutes after swarming activity started, swarm size was estimated by eye. During swarming time, mating pairs were collected using insect sweep nets and placed into individual labelled cups. Then, the origin of males (laboratory or wild) in each mating pairs was determined under a binocular microscope according to the colour of the fluorescent powder on each mosquito. Female insemination status was assessed as previously described. Ten replicates were conducted for each mating combination. For each replicate, colour marking and mating combinations were randomly assigned.

### Experiment 3: Swarm characteristics and male competitiveness of three wild populations

In this experiment, only F1 wild mosquitoes from Bama, Soumousso or Kongoussi were used. As previously described, 3 to 4-day-old virgin males and females were released at sunset into compartments following a sex ratio of 3:1 (male:female) according to six mating combinations: (a) 300 Bama males + 100 Bama females; (b) 300 Kongoussi males + 100 Kongoussi females; (c) 300 Soumousso males + 100 Soumousso females; (d) 100 Bama males + 100 Kongoussi males + 100 Soumousso males + 100 Bama females; (e) 100 Bama males + 100 Kongoussi males + 100 Soumousso males + 100 Kongoussi females and (f) 100 Bama males + 100 Kongoussi males + 100 Soumousso males + 100 Soumousso females. Most of the time, mixed swarms were characterized by more than one centroid (up to three rarely). As the smaller centroids were unstable and had a low male density, only the largest centroid for each swarm was recorded and swarm size was estimated by eye 15 min after swarming activity started As before, males were marked according to their origin, mating pairs were collected and identified, and female insemination status was assessed. Ten replicates were performed for each mating combination. For each replicate, marking and mating combinations were randomly assigned.

All experiments were performed over two years during the rainy season. Experiments 1 and 2 were carried out between June and October 2016 and while the Experiment 3 was carried out between July and October 2017.

### Statistical analyses

All analyses were performed using R (*version* 4.0.2; https://cran.r-project.org). Generalized Linear-Mixed Models (GLMM, lme4 package) were used in all analyses with both replicates and observers (confounded with the compartment and the swarm identity) considered random effects.

In *Experiment 1*, swarm size (i.e. the estimated number of males in a swarm) was analysed with a negative binomial distribution to account for overdispersion. Male origin (2 levels: wild or laboratory) was considered a fixed effect. Swarm height was analysed with a Gaussian distribution after data transformation (powerTransform function in the library car). Male origin, the number of swarming mosquitoes and their interactions were considered fixed effects. The number of mating pairs was analysed with a Gaussian distribution. The swarm height, mating combinations, number of swarming mosquitoes and their interactions were considered fixed effects. The insemination rate was analysed with a binomial distribution and mating pair identity was considered fixed effect.

In *Experiments 2 and 3*, the number of mating pairs was analysed with a negative binomial and a Gaussian distribution, respectively. Swarm size, swarm type (2 levels: "pure" when males of a single origin were released or "mixed" when males of several origins were released into the same compartment), female origin (2 levels in experiment 2: laboratory or wild; and 3 levels in experiment 3: Bama, Soumousso or Kongoussi), swarm type * female origin and swarm type * swarm size interactions were considered as fixed effects. To assess impact of female mating choice the on number of mating pairs, a data subset was used including only mixed swarms. A Gaussian distribution was used with female origin, male origin and their interactions considered fixed effects. The insemination rate was analysed with binomial distributions. Swarm type, mating pair identity (i.e. the origin of both the male and the female of the mating pairs, 4 and 9 levels in Experiments 2 and 3, respectively) and their interactions were considered fixed effects. In Experiment 3, swarm height of the different mosquito populations was analysed with a Gaussian distribution on power transformed data. Swarm type (4 levels: mixed swarms, pure Bama, pure Soumousso, pure Kongoussi), swarm size and their interaction were considered fixed effects.

For model selection, we used stepwise removal of terms, followed by likelihood ratio tests. Term removals that significantly reduced explanatory power (*P* < 0.05) were retained in the minimal adequate model^52^. Residuals of the minimal models were checked for a normal distribution and for homogeneity of variances with Shapiro and Fligner tests, respectively. The post-hoc Tukey method was used for multiple comparison using the ‘glht’ function of the package ‘multcomp’ when necessary^53^. All means are provided with their standard error and percentages expressed with their 95% confident interval.
